# The efficacy and safety of Jinhua Qinggan granule (JHQG) in the treatment of coronavirus disease 2019 (COVID-19): A protocol for systematic review and meta analysis

**DOI:** 10.1097/MD.0000000000020531

**Published:** 2020-06-12

**Authors:** Qiongshuai Zhang, Fang Cao, Yufeng Wang, Xiaohong Xu, Yihan Sun, Jiannan Li, Xun Qi, Shaoqian Sun, Guangcheng Ji, Bailin Song

**Affiliations:** a Department of Acupuncture and Tuina, Changchun University of Chinese Medicine, Changchun; b Department of Acupuncture, The First affiliated Hospital of Henan University of TCM, Zhengzhou; c Department of Tuina, Traditional Chinese Medicine Hospital of Jilin Province; d Graduate school; e Department of TCM, Changchun University of Chinese Medicine; f Department of Rehabilitation, Chian-Japan Union Hospital of Jilin University; g Department of Rehabilitation, The Third Affiliated Hospital of Changchun University of Chinese Medicine, Changchun 130117, China.

**Keywords:** meta-analysis, systematic review, Jinhua Qinggan granule, COVID-19, protocol

## Abstract

**Background:**

Currently, the global number of infected novel coronavirus has exceeded 2.6 million and the death toll has exceeded 170,000, but the specific drug for the treatment of COVID-19 has been not appears. In the process of fighting COVID-19 in China, JHQG has been promoted by the Chinese government and widely used in the treatment of COVID-19. The purpose of this study is to systematically evaluate the efficacy and safety of JHQG for COVID-19.

**Methods:**

We are going to search the electronic databases: PubMed, EMBASE, Cochrane library, Web of Science (WOS), Google scholar, China National Knowledge Infrastructure (CNKI), Chinese Biomedical literature Database (CBM), Chinese Scientific and Journal Database (VIP), Wan Fang database (Wanfang) for published clinical trails and search clinical trials register platforms of Chinese Clinical Trial Registry (ChiCTR) and ClinicalTrials.gov (www.ClinicalTrials.gov/) for ongoing trials of Jinhua Qinggan granule for COVID-19. The primary outcomes of the included studies contain Clinical symptom disappearance rate and the secondary outcomes obtain: TCM syndrome scale score, Hamilton anxiety scale score, and adverse events. We will use RevMan V5.3 software to perform the calculations. PRISMA-P checklist was used in writing this report.

**Results:**

The study results will be submitted to a peer-reviewed journal for publication.

**Conclusion:**

This study will provide a high-quality evidence of the efficacy and safety of Jinhua Qinggan granule on patients with COVID-19.

**PROSPERO registration number:**

CRD42020181919.

## 1 Introduction

In December 2019, COVID-19 caused by severe acute respiratory syndrome coronavirus 2(sars-cov-2) was first discovered in Wuhan China.^[1–3]^ The virus was found to be highly infectious and susceptible to infection in all kinds of people. At present, the epidemic has spread rapidly, with more than 2.6 million COVID-19 cases confirmed globally,^[4]^ which is seriously threatening human life and health. On March 12, 2020, the WHO declared the COVID 19 outbreak to be characteristically a pandemic. Symptoms of COVID-19^[5–8]^ mainly include cough, fever, expectoration, dyspnea, chest pain, fatigue, loss of appetite, headache, myalgia, hemoptysis, and diarrhea. As a new viral infectious disease, COVID-19 still can not be treated with specific therapeutic drugs.^[9]^ A recent study^[10]^ found that among the confirmed patients, 69.9% of patients were diagnosed with mild pneumonia, 25.5% with severe pneumonia, patients with severe condition always to be the old male. Another study^[11]^ of 41 confirmed COVID 19 inpatients showed similar results: the majority of infections were male (30 out of 41 cases, 73%).

During the fight of COVID-19 epidemic in China, it was found that Chinese herbal medicine had a good effect on COVID-19. Chinese central government had formulated series of TCM diagnosis and treatment plans for COVID-19,^[12–16]^ and recommended a batch of Chinese herbal medicines for the prevention and treatment of COVID-19.^[15,16]^ JHQG is one of them. However, there has been no systematic review of JHQG for COVID-19. Therefore, in this study, the efficacy and safety of the application of JHQG to COVID-19 will be systematically evaluated.

## 2 Methods and analysis

We used Preferred Reporting Items for Systematic Review and Meta-Analysis Protocols (PRISMA-P) 2015 statement^[17]^ in writing this report.

### 2.1 Study inclusion criteria

Studies of Randomized controlled trials (RCTs) with JHQG for COVID-19 will be included. There won’t be any restriction on publishing language and publishing status. Patients diagnosed with COVID-19 of all ages. Genders and racial groups will be included. The intervention of the experimental group should contain JHQG alone or with other interventions. While, the control group could use any other treatments (e.g., External therapy, usual or standard care, placebo, or no treatment) except JHQG. Primary outcomes of the included studies contain Clinical symptom disappearance rate. The secondary outcomes obtain: TCM syndrome scale score, Hamilton anxiety scale score and adverse events.

### 2.2 Search methods

We will search the following electronic databases of PubMed, EMBASE, Cochrane library, Web of Science (WOS),Google scholar, China National Knowledge Infrastructure (CNKI), Chinese Biomedical literature Database (CBM), Chinese Scientific and Journal Database (VIP), and Wan Fang database (Wanfang) for identifying literature of studies of JHQG for COVID-19, the search strategy of Pbumed will be shown as Table 1, we would also change the search strategy properly according to exact database. Unpublished data of Ongoing trials will be searched from Chinese Clinical Trial Registry (ChiCTR) and ClinicalTrials.gov (www.ClinicalTrials.gov/). All the databases and online registration platforms will be searched from inception to May 30, 2020 and there will be no language restrictions.

**Table 1:**
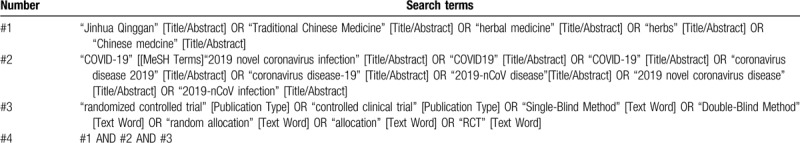
Search strategy for PubMed.

### 2.3 Data collection and management

#### 2.3.1 Selections of studies

**Figure 1:**
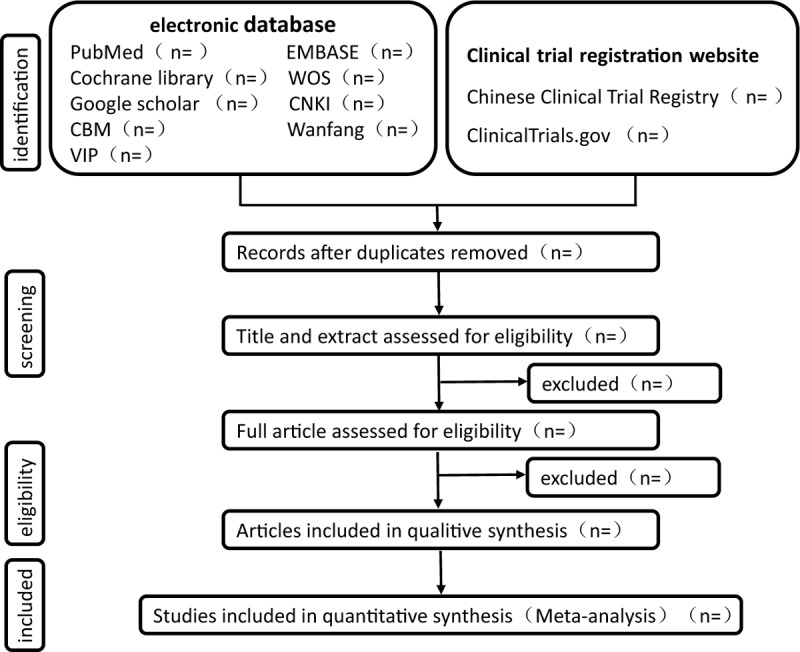
The screening process.

Two reviewers (CF and XQ) will import the retrieved literature into Endnote software (V. X9.0), they will screen literature by inclusion and exclusion criteria independently. Firstly, duplicate literature will be eliminated by Endnote, and then the obviously inappropriate ones will be eliminated by reading the title and abstract. Finally, the last inappropriate literatures will be eliminated by reading the full article. (Fig. 1 shows the screening process) If it comes with disagreements, which will be arbitrated by a third reviewer (JNL)

#### 2.3.2 Data extraction

Two reviewers (CF and XQ) will review all the included studies and extract data contain items of title, first author, publication year, country, publication language, journal, information of participants: gender, age, study design, sample size, intervention, controls, type of measures, primary and second outcomes and other detail information. All the data, which will be cross-checked by the 2 reviewers, would be extracted with Excel (V.2019) software. The results will be cross-checked by the 2 reviewers.

### 2.4 Risk of bias assessment

Two reviewers (QSZ and FC) will assess the methodological quality of the included studies with the Cochran collaboration tool. The bias of sequence generation, allocation concealment, blinding of participants personnel and outcome assessment, incomplete outcome data, selective outcome reporting, and other bias will be accessed by the 2 authors. A third reviewer (BLS) will arbitrate the disagreement which appears in the evaluation process. The risk of bias would be classified^[18]^ as low, high, or unclear.

### 2.5 Measures of treatment effect

To assess JHQG in the treatment of COVID-19. We will use relative risk (RR) for dichotomous data. Mean differences (MD) or the standard mean differences (SMD) for continuous data. Using 95% confidence intervals (CI) to show the effect sizes.

### 2.6 Dealing with missing data

Two reviewers (GCJ and XQ) will try to contact the corresponding author for missing or insufficient by e-mail or telephone. We will perform the analysis based on the available studies, if we failed to get the data missed. We would also evaluate the potential impact of missing information on the outcome.

### 2.7 Assessment of heterogeneity

Statistical heterogeneity of included studies will be assessed with a standard *I*^2^ test, if *I*^2^ < 50%, the heterogeneity of the texts may be ignored, the fixed-effect model will be applied. While if *I*^2^ ≥ 50%, statistical heterogeneity will be regarded as significant, the random-effects model will be used.

### 2.8 Assessment of reporting bias

We will make use of funnel plot to assess the reporting bias with more than 10 studies are included.^[19]^ If the funnel is symmetrical, which indicates there is no publishing bias, otherwise, there is. But if the included studies are less than 10, *P* value will be used.

### 2.9 Data syntheses

We will make use of RevMan (version 5.3) software to conduct the meta-analysis. Fix-effect model will be applied with the condition that there is no heterogeneity of the results. Otherwise, we will turn to the random effects model after the clinical heterogeneity has been taken out.

### 2.10 Analysis of subgroups or subsets

Subgroup analysis will be conducted based on gender, age, hospitalization time, or other conditions of participants, if potential heterogeneity exists in the included studies.

### 2.11 Sensitivity analysis

We would conduct a sensitivity analysis to assess the robustness of the study results. We will focus on the processing method of missing data, sample size, and methodological quality.

### 2.12 Grading the quality of evidence

We will take advantage of Grading of Recommendations Assessment, Development and Evaluation Reliability Study (GRADE) to assess the quality of evidence. The grades are very low, low, moderate, and high.

### 2.13 Ethics and dissemination

Ethical approval is not needed for this systematic review. For nothing of the data in this review is related to an individual patient.

## 3 Discussion

COVID-19 is wreaking havoc around the world. JHQG is a traditional Chinese herbal medicine prescription, which was recommended by the National Health Commission of China in the Plan of Diagnosis and Treatment for COVID-19.^[13–16]^ JHQG has been made extensive use in the treatment of COVID-19 in China, recent studies shows that JHQG has good effect on the treatment of COVID-19, However, the mechanism is not clear, health authorities in countries other than China may have doubts about the effectiveness and safety of JHQG. Thus, a systematic review about it is urgently needed. This study could provide evidence of JHQG used in the treatment of COVID-19 and help the clinicians to make decisions.

## Author contributions

QSZ, YFW, BLS and FC designed this review. JNL, XQ, and XHX contributed to developing the search strategy and drawing the figure of the study selection process. QSZ, YHS, GCJ, and SQS wrote the manuscript. All authors approved the final version of the manuscript.

**Data calculation:** Guangcheng Ji, Xun Qi.

**Formal analysis:** Qiongshuai Zhang, Fang Cao, Yufeng Wang, Guangcheng Ji, Bailin Song.

**Funding acquisition:** Bailin Song.

**Resources:** Qiongshuai Zhang.

**Software:** XunQi, Jiannan Li.

**Writing – original draft:** Qiongshuai Zhang, Yihan Sun, Guangcheng Ji and Shaoqian Sun.

**Writing – review and editing:** Bailin Song, Yufeng Wang
